# Analysis of a summary network of co-infection in humans reveals that parasites interact most via shared resources

**DOI:** 10.1098/rspb.2013.2286

**Published:** 2014-05-07

**Authors:** Emily C. Griffiths, Amy B. Pedersen, Andy Fenton, Owen L. Petchey

**Affiliations:** 1 Department of Entomology, North Carolina State University, Raleigh, NC 27695-7613, USA; 2 Department of Animal and Plant Sciences, University of Sheffield, Alfred Denny Building, Western Bank, Sheffield S10 2TN, UK; 3 Centre for Immunology, Infection and Evolution, Institute of Evolutionary Biology, School of Biological Sciences, Ashworth Labs, University of Edinburgh, Kings Buildings, West Mains Road, Edinburgh EH9 3JT, UK; 4 Institute of Integrative Biology, University of Liverpool, Liverpool L69 7ZB, UK; 5 Institute of Evolutionary Biology and Environmental Studies, University of Zürich, Winterthurerstrasse 190, Zürich 8057, Switzerland

**Keywords:** degree distribution, ecological network, indirect interactions, modularity, parasite ecology, polymicrobial infection

## Abstract

Simultaneous infection by multiple parasite species (viruses, bacteria, helminths, protozoa or fungi) is commonplace. Most reports show co-infected humans to have worse health than those with single infections. However, we have little understanding of how co-infecting parasites interact within human hosts. We used data from over 300 published studies to construct a network that offers the first broad indications of how groups of co-infecting parasites tend to interact. The network had three levels comprising parasites, the resources they consume and the immune responses they elicit, connected by potential, observed and experimentally proved links. Pairs of parasite species had most potential to interact indirectly through shared resources, rather than through immune responses or other parasites. In addition, the network comprised 10 tightly knit groups, eight of which were associated with particular body parts, and seven of which were dominated by parasite–resource links. Reported co-infection in humans is therefore structured by physical location within the body, with bottom-up, resource-mediated processes most often influencing how, where and which co-infecting parasites interact. The many indirect interactions show how treating an infection could affect other infections in co-infected patients, but the compartmentalized structure of the network will limit how far these indirect effects are likely to spread.

## 1. Introduction

More than 1400 parasite species, including viruses, bacteria, helminths, protozoa and fungi, infect humans [1]. Simultaneous infection of humans by multiple species (co-infection) is commonplace [2–4]; helminth co-infection alone affects 800 million people [5]. Co-infection involves globally important diseases such as HIV and tuberculosis [6], is concentrated among the poor [7,8] and is often associated with worse host health and higher parasite abundance than hosts with single infections [9]. Co-infection can also reduce treatment efficacy [10–12] and increase treatment costs [13]. These phenomena are likely driven by interactions among co-infecting parasites [14].

Species, including co-infecting parasites, interact when individuals of one species affect individuals of another [15]. Such interactions among co-infecting parasites, host tissues and the immune system can be viewed as a network [16]. Interactions between parasites in this network may be direct [17], or indirect mediated by other parasite species, host immunity [3,18,19] or resources [20,21]. Parasites consume resources by eating and inhabiting parts of their host [22]. When interactions occur, treatment of one species could result in changes to another parasite not directly targeted by the treatment [19,23,24]. However, we do not know the frequency of ‘bottom-up’ resource-mediated or ‘top-down’ immune-mediated interactions among parasites [16], or how they are modified by the introduction of new parasites [25], despite considerable biological interest in the topic. Indeed, the potentially overwhelming diversity of co-infecting parasite types, and their many possible interactions, means that understanding the consequences of co-infection for human health and parasite dynamics remains difficult.

Before the effects of treatment on co-infecting parasite dynamics can be accurately predicted, we need to know how within-host parasite communities are structured. If parasite communities have consistent, non-random assembly processes, then these could be used to develop general treatment guidelines. However, at present, we do not know the overall structure of the wider parasite community of humans, because most studies of co-infection are typically restricted to measuring interspecific interactions between pairs of parasites (80% of publications reviewed in reference [9] reported a single species pair, e.g. [26–28]). Here, we move beyond this pairwise view to study the potential interactions among the many parasites that can co-infect humans. We do this by assembling a summary network.

Network structure reveals aspects of the biological function and stability of complex systems [29,30], and networks have frequently been used to study free-living ecological communities, in the form of food webs of feeding relationships. Summary networks are built from relationships observed across multiple places and times, and are particularly useful for identifying general forces influencing community composition, even when they are not directly measured from a single sample [31,32]. For example, a summary network could show all the feeding interactions observed in a freshwater stream through gut contents analysis of many individuals sampled at different times [33], allowing prediction of possible community responses to invasion of new species. Applying similar principles to within-host parasite co-infection networks, one can take reported relationships between two co-infecting parasites and use them to extrapolate to possible relationships with other parasites were such co-infections to occur. For example, if hepatitis viruses compete for liver cell resources [34], there is potential for another liver-consuming parasite such as *Fasciola hepatica* to compete with them, were co-infection between a liver fluke and hepatitis virus to occur. Similarly, microparasites and macroparasites might interact via immune components such as T-helper cells [3,16]. Researchers have begun to include parasites in food webs for particular ecosystems such as estuaries [35,36], in disease transmission networks [37], in networks of comorbidities [38] and in summary networks of parasites across fish species [39]. Networks of within-host ecosystems have also revealed interactions within hosts involving *Mycobacterium tuberculosis* infection [40] and microbial communities [41]. However, to the best of our knowledge, there has been no attempt to construct a summary network of interspecific parasite interactions in a single host species.

We constructed a summary network for human co-infections, with three within-host trophic levels, to find out whether interactions among parasites tend to be direct or indirect, or are predominantly resource-mediated or immune-mediated. The summary network documents all the co-infecting parasites and related parts of human physiology, akin to many ecological networks of free-living systems that aggregate all ecological interactions in one ecosystem type. Hence, the summary network of human co-infection presented here does not represent an individual co-infected host, but reflects potential interactions reported among the parasite community within humans.

**Figure 1.**
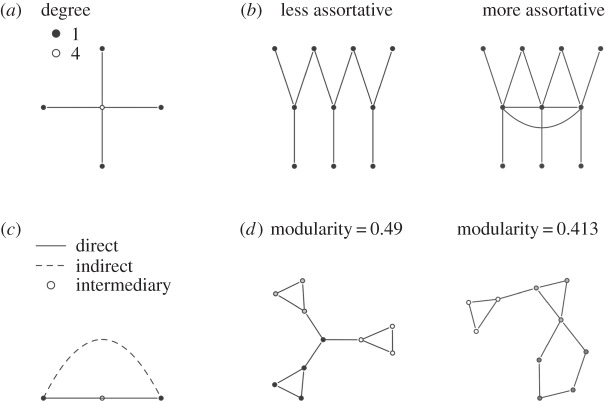
Illustrative diagrams of network analyses undertaken: (*a*) node degree, (*b*) assortativity, (*c*) direct and indirect connections and (*d*) modularity. Left network in (*d*) was designed to have three modules and high modularity; right network in (*d*) is a random network with the same number of nodes, links and modules, but lower peak modularity.

Networks are composed of nodes and links between pairs of nodes. The network we construct has three types of node: parasites (e.g. HIV, *Aspergillus*, hookworm), host immune system components (e.g. IgA, IL-10, macrophages) and host resources (including nutrients or cells consumed and cells, bodily fluids, tissues, organs, anatomic sites inhabited or damaged by parasites). We analysed (i) the structure of the network in terms of the distribution of reported interactions between nodes, (ii) the frequency of parasite interaction types (direct, immune-mediated, resource-mediated or parasite-mediated) and (iii) whether the network is arranged in modules of highly connected nodes (table 1 and figure 1). We found that the entire network comprised several discrete submodules and was dominated by indirect links between parasites, and that these interactions among parasites arose mainly through ‘bottom-up’ control.

**Table 1.** Network metrics used herein and their relevance to interactions among co-infecting parasites.

measure	meaning	importance to co-infection	outline
degree	number of nodes linked to a given node	reveals how interactive a node is	figure 1*a*
assortativity	correlation of node degree across all pairs of linked nodes	strong positive correlation indicates polarization between nodes with few and many links; cliques of highly interactive nodes may need special treatment	figure 1*b*
direct parasite interactions	number of parasites linked to a given parasite	reveals co-infections where integrated treatment may be advisable	figure 1*c*
indirect parasite interactions	number of parasites connected to each parasite by two links via an intermediary node	reveals interactions between co-infecting parasites mediated by another parasite or by host immunity or resources, where treatment choice may depend on host condition	figure 1*c*
modules	groups in the network with many internal links and fewer links out to other groups	reveals areas of highly connected immune components, parasites and resources; could enable typing of co-infection cases	figure 1*d*

## 2. Methods

We assembled a network of parasites, their resources and immune components from 316 articles on human hosts with established co-infections published in 2009 (see reference [9] for inclusion/exclusion criteria). Because we found our results robust to number of publications sampled within 2009, we assume they would be robust to sampling more publications from other years (the electronic supplementary material (ESM), figures S1 and S2). Each publication reported the resource and immune interactions most relevant to that study, such as the interactions involved in HIV–tuberculosis co-infection, but did not report information on potential interactions beyond that. To understand the wider niche of the parasites, we therefore combined links from many such publications into a single summary network.

An interaction is denoted by a link between two nodes (resource, parasite or immune components). All links in the network were binary (present or absent). We did not assign interaction strength to the links, because requisite data were unavailable from most publications, and a binary network still reveals the topology of biotic interactions (see reference [9] for fuller discussion of the difficulty of quantifying interaction strengths from this dataset). Some networks assign directions to links. However, the presence of many links where the direction was indeterminate (e.g. non-mechanistic links between parasites, immune interdependencies, ambiguity in the source publication), and the inability to analyse a network with a mixture of directed and undirected links means all three versions of the network presented here (see below) were wholly undirected. None of the metrics we used depends on link direction (metrics discussed in §2*a*–*d*).

In the published studies, nodes described in different ways may have referred to the same biological component. For example, ‘digit’ and ‘finger’ can both refer to an appendage on one's hand. To detect functionally similar links and following standard practice in network science [42], we aggregated closely related nodes, so they had the same name. Following common use in genetics, we used an ontology [43],^1^ the Universal Medical Language Service (UMLS) semantic hierarchy^2^ and the following rules, to ensure consistent node aggregation: (i) immune and resource nodes aggregated to cell type or above, except for components that interact directly with parasite, (ii) nodes designated in the UMLS as substances were aggregated by biological function and (iii) nodes of the human reproductive system were classified into gender-specific classes (e.g. female genitals, male genitals and pregnancy), because differences between the sexes and reproductive status have been important in other co-infection studies [44–46]. Accordingly, some nodes above the cellular level were subsets of one another, such as knee and joint, gums and mouth or colon and gastrointestinal. These nodes were not aggregated so as not to confound how link patterns were counted. For example, the number of indirect links between parasites will increase as intermediary nodes are aggregated. (An indirect link between two nodes occurs when two nodes are connected via a third node.) Relations between such nested nodes (such as colon and gastrointestinal) are biologically important, and the module analysis allows these nodes to cluster together. Because the amount of node aggregation can affect network structure [47], we assessed the sensitivity of our conclusions to: (i) no aggregation, where node names matched those reported in the publications; (ii) medium aggregation of cells into tissues, immune receptors into functional groups and parasites to genus level; (iii) high aggregation where resource or immune nodes were aggregated into body parts, and parasites were aggregated to the family level.

Links between nodes were first derived from the same publications that reported interactions among the nodes concerned. For parasites where resource or immune links were not reported in the publications, we allocated links with reference to a comprehensive infectious disease encyclopaedia [48]. Each link was classified in one of three ways according to the strength of evidence: (i) co-occurrences (two nodes observed in the same individual), (ii) correlations (an association between two nodes is reported, without a known biological mechanism) or (iii) mechanistic links (connected by a demonstrated biological process). While known mechanisms are a reliable basis for including a link in the summary network, there are potential causal processes that remain unknown, especially for poorly studied parasites or where experimentation on human subjects is precluded. Two components found simultaneously in the same individual could potentially interact, even if the interaction is weak or the mechanisms have not been identified. Therefore, three versions of the network were analysed based on the above-described link types: mechanistic links only, mechanistic and correlative links, and all three link types together. These three versions span from a network with high degree of certainty (mechanistic only) to one where the associations and mechanisms have not been reported (all link types).

### (a) Network analysis

We analysed three structural features of each of the three versions of the network (figure 1 and table 1): (i) how the components are linked (figure 1*a,b*), (ii) the frequency of different links among parasites (figure 1*c*) and (iii) whether the network contains modules of tightly linked nodes (figure 1*d*). Other features can be studied, but we chose these ones because they reveal functionally important patterns of interactions in co-infected humans (table 1). Analyses were done in R v. 2.15.1 [49].

### (b) Degree distribution

A node's degree is the number of nodes that are one link away. A network's degree distribution reveals how links are distributed among nodes, can indicate how resistant the network is to perturbation and, being a commonly used network metric, enables us to directly compare the within-host co-infection network with others [50]. We estimated the parameter(s) for exponential, power-law, Poisson, normal and uniform distributions using maximum likelihood, and calculated the coefficient of determination (*R*^2^) to find the fitted distribution closest to the observed degree distribution [51]. We also analysed the tendency for well-connected (high degree) nodes to be linked to other well-connected nodes (evidence of assortativity). Assortativity was measured via Pearson's correlation coefficient (*r*) for the degree of nodes either end of each link [50, §3.6, pp. 192–193]. Networks with high assortativity have high positive values of *r* (close to +1), because high degree nodes are also likely to be linked to other high degree nodes, giving greater potential for perturbations to spread across the network [52]. Negative values of *r* indicate disassortativity whereby high degree nodes are dispersed across the network and are typically connected to low degree nodes.

### (c) Direct and indirect interactions

Interactions are indirect when two parasite nodes are linked via a single intermediate node (either a resource, parasite or immune component). Direct interactions have no intermediary. We counted the number of these interactions between every pair of parasites in the network. We compared these totals with that expected from chance using 1000 randomly rewired networks containing the same number of links as the observed network. We used a constrained null model of a simple Poisson process, so there was the same number of nodes in each trophic level, but each node had equal probability of being linked to another node (independent link assignment, following [30,53]). Most biological networks deviate from this null distribution, but we use it because researchers have argued that parasite community assembly is a neutral, independent process [54]. More constrained models could be tested in future (e.g. scale-free networks [30]), but as this is the first summary network of parasites within humans, we begin with a simple Poisson distribution of links. We used a normal distribution to calculate the probability of the observed number of links from our randomization, because a Poisson distribution with large mean approximates a normal distribution.

### (d) Modules

Modules were found using three search algorithms: (i) sequentially removing the most peripheral link [55]; (ii) using statistical mechanics (the methodology of [56], iterated 100 times); and (iii) using short random walks [57]. These algorithms search for groups of nodes (modules) that maximize modularity, and we compare the results of all the identified module sets from all three algorithms to find the set with highest modularity (ESM, figure S1*a*). The algorithms varied in the final measure of modularity, but visually comparing the modules in each module set revealed many components repeatedly co-occurring. We used three search algorithms to give a better chance of finding the optimal grouping of species in modules than would have been achieved using a single search algorithm. One measure of modularity, termed *Q*, ranges from 0 (no modular structure, many links between modules) to 1 (strong modular structure, few links between modules) [55]. We analysed the set of modules with peak modularity (*Q*) for the mechanistic network, because this version of the network makes a conservative assumption about the presence of interactions and likely reveals the strongest functional patterns within the network. For each module, we recorded the type (parasite, resource, immune) and identity of the node with highest within-module degree. These nodes contribute strongly to modularity and reveal the defining characteristics of each module (table S1 and figure S3).

We also tested whether modules had more within-module links than expected by chance. We repeated this test for two link types (immune–parasite and resource–parasite). We ignored direct parasite-to-parasite links, because these were rare in the mechanistic network. The number of observed links of a particular type was considered different from expected if it lay beyond either tail of a binomial distribution (i.e. *p* < 0.025 or *p* > 0.975). The *p*-value was calculated given a binomial distribution with the number of trials being the total number of links of that type in that module, and the probability of success being the proportion of nodes of that type in that module. We also examined whether resource-dominated modules were also present in four alternative module sets with next-highest *Q*-values, where 0.4690 < *Q* < 0.4695).

## 3. Results

The summary network of co-infected humans comprised 124 host resources, 305 parasite taxa, 98 immune system components and 2922 links between these components. Most publications (256/316, 81%) reported data from multiple patients. The majority of links (1578) were based on mechanistic evidence, whereas 812 were from co-occurrence, and 532 from correlational evidence. We primarily describe results for the mechanistic-only version, because these links have greatest biological support. We compare these with other network versions with less mechanistic support to show the range of potential interactions.

### (a) Degree distribution

**Figure 2.**
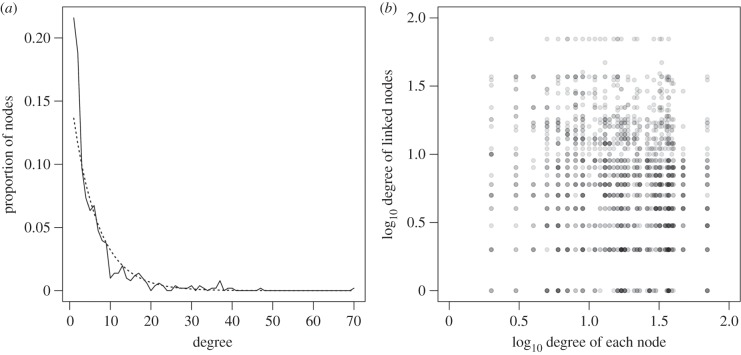
(*a*) Raw degree distribution for the mechanistic network. Solid line is the observed proportion of nodes with a degree greater than or equal to the value on the *x*-axis. Dashed line is the best-fitting statistical model (exponential model *λ* = 0.16, *p* < 0.001, *R*^2^ = 0.87). (*b*) Assortativity: the degree of each node plotted against the degree of their linked nodes for all unique links for the mechanistic network (Pearson's correlation *r* = −0.12, *p* < 0.001). Plotting symbols are transparent such that 10 overlaid data points are black.

The degree distribution of the mechanistic network most closely resembled an exponential distribution with the exponent 0.16 (s.d. 0.007, *R*^2^ = 0.87, *p* < 0.001; figure 2*a*). This means that most nodes (i.e. parasites, resources or immune components) in the network were linked to few other nodes; in fact, 89.7% of nodes (456/508) had fewer than 15 unique links. Only nine nodes (0.018%) had degree greater than or equal to 35. These highly connected nodes were blood (70 unique links), respiratory tract (47), skin (40), lungs (39), HIV (37), IgG (37), macrophage (37), dental abscess (37) and liver (36). There was generally weak assortativity in all three versions of the network (*r* close to zero, ranging from −0.12 to 0.12; the ESM, table S2 and figure S4), although there was significant disassortativity in the mechanistic network (*r* = −0.12, *p* < 0.001, figure 2*b* and ESM, table S2).

### (b) Direct and indirect parasite interactions

**Figure 3.**
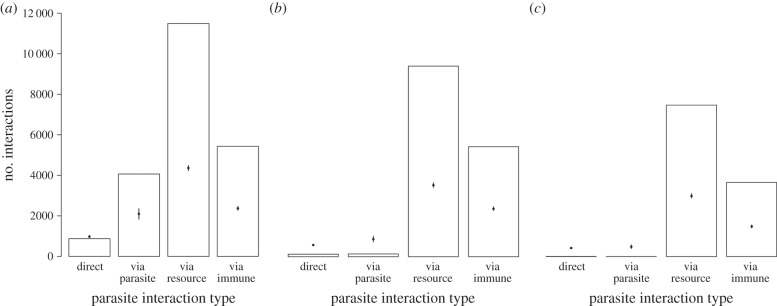
The number of direct and indirect paths between parasites for (*a*) all link types, (*b*) mechanistic and correlative links and (*c*) mechanistic links only. Vertical black lines represent expected distributions (2 s.d., dot indicates mean) from 1000 simulations. All observed results deviated significantly from expected values (tested against normal distribution, *p* < 0.001). Vertical axis scales for (*a–c*) are identical.

Indirect interactions between parasites were more common than direct links. The ratio of indirect to direct links ranged from 1.09 times higher for parasite-mediated interactions within mechanistic and correlative link networks, to 829 times higher for resource-mediated interactions in the mechanistic-only network (figure 3 and ESM, table S2). Indirect parasite interactions were most often resource-mediated, and these were significantly more common than expected by chance (*p* < 0.001; rewiring randomization test). Immune-mediated indirect interactions were about half as common as resource-mediated interactions, though still significantly more common than expected by chance (*p* < 0.001). Furthermore, 167 publications (53%) contributed multiple parasite–resource links, but only 85 (27%) contributed multiple parasite–immune links. The relative frequency of reported resource- and immune-mediated interactions was robust to the potential under-reporting of parasite–immune links (ESM, figure S5), and to the exclusion of publications relating to individual patients (ESM, figure S6). Most parasite-only links were based on co-occurrence; networks excluding this type of evidence had relatively few direct or indirect interactions involving only parasites (and fewer than expected by chance; *p* < 0.001; figure 3*b,c*). The relative frequency of parasite-only links was qualitatively similar in all three networks (figure 3*a–c*, all *p* < 0.001; ESM, table S2).

### (c) Modules

In the mechanistic network, 10 modules were detected, ranging in size from 12 to 90 nodes (peak modularity was 0.4695; ESM, table S1 and figure S1*a*). We visually compared the nodes in each module in these other high modularity sets with the 10 modules described above and confirmed that all modules were consistently associated with bodily locations and that the node with highest degree was often a resource. Each module contained a mix of immune components, resources and parasites (except one module, which contained only bacteria). Parasites were the most common node in nine of the 10 modules (ESM, table S1, except module 2 with 30 immune and 22 parasite nodes). All but two modules had more resource than immune nodes (module 2 had 30 immune and 15 resource nodes, and module 4 had 25 immune and nine resource nodes). These 10 modules were associated with particular microhabitats within the human body (ESM, figure S3 and table S1), and this association was also found in other module sets with next-highest modularity values (results not shown). Visual inspection of these 10 modules showed associations with particular bodily systems (modules 3, 4, 7, 9; numbers refer to ESM, table S1), body parts (modules 1, 8, 10) and tissues (module 6). Two modules were classified as mixed because they contained several sites of infection, including the oesophagus, genitals and eyes (module 2), and nose, skin and urinary tract infections (module 5).

**Figure 4.**
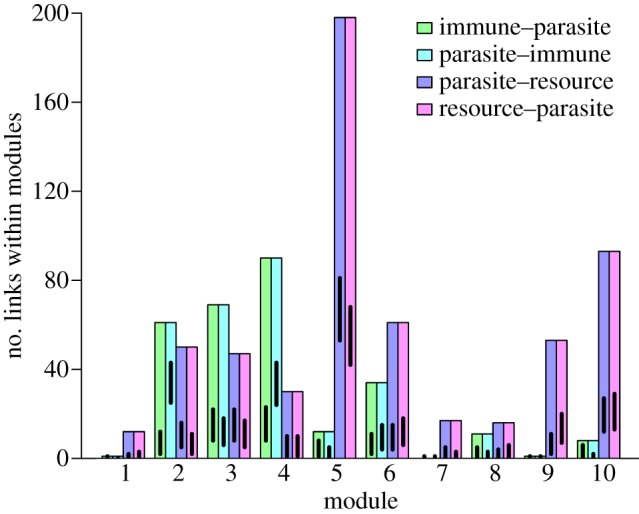
Number of within-module links between host immune components and parasite and between host resources and parasites in each of the 10 modules of the mechanistic network. Lines indicate 95% confidence intervals from the binomial test. Bars overlapping with lines (immune–parasite links for modules 1, 7 and 9) are within expectations (*p* > 0.05). There are more within-module links for all other modules and link types than expected (*p* < 0.001).

Resource nodes had the highest within-module degree for seven of the 10 modules, and were more common than expected by chance in all modules (figure 4, *p* < 0.001). Parasite–immune links dominated the structure of the remaining three modules, where they were also more common than expected by chance (*p* < 0.001). Of the three modules where non-resource nodes had the highest within-module degree, two were immune nodes (IgG and macrophages), and a parasite (HIV) dominated the other.

### (d) Robustness of results

We tested whether our measures of network structure were sensitive to the aggregation of nodes and the publications used (ESM, figures S1*b* and S2 and tables S3–S5). The key findings of exponential degree distributions (ESM, figure S7), weak (dis)assortativity (ESM, figure S8), the relative frequency of parasite interaction types (ESM, figure S9) and resource-mediated outnumbering immune-mediated within-module interactions (ESM, figure S10) were robust to node aggregation. While the number of nodes and links in the network increased linearly with each new publication (ESM, figure S1*b*), the ratio of resource- to immune-mediated interactions levelled off once 40 publications were sampled, with resource-mediated interactions being dominant (ESM, figure S2*a*). The degree distribution exponent also reached an asymptote after 100 publications, but the *R*^2^-value was unchanged even with only five publications sampled (ESM, figure S2*b*). Assortativity was weakly positive with a very low *p*-value, and reached an asymptote after 100 publications (ESM, figure S2*c*). The number of modules and the modularity score peaked once 50 publications were sampled, and levelled off at lower values with fewer modules and more sampling (ESM, figure S2*d*). We also tested whether a bipartite version of the network with host–parasite links was nested: it was not (ESM, figure S11).

## 4. Discussion

We developed a summary network of human co-infection from published reports of co-infecting parasites, the resources they consumed and immune reactions to them. The summary network was complex, but contained several clear structural patterns. First, most components were linked to few other components, although some parasite species were highly interactive, e.g. HIV, *Staphylococcus aureus* and hepatitis C virus each interacted with dozens of other nodes. Second, most pairs of parasites were linked indirectly. While many studies highlight immune-modulation by parasites [58–60], we found twice as many pairs consuming the same resource as sharing immune responses. Finally, links were clustered around particular locations of the human body, suggesting that the parasite community may be divided into microhabitat modules.

These findings indicate that the human summary co-infection network has many features in common with free-living community networks, confirming prior suggestions that co-infection can be understood using ecological concepts [16,61]. First, assortative and disassortative processes were found (ESM, tables S2–S4), similar to directed ecological networks [62,63]. This suggests that, while well-connected parasite species tended to interact with one another, other well-connected resource and immune nodes tended to interact with poorly connected components. This may have limited how far perturbations are likely to spread across the network [52]. Second, the observed exponential degree distribution matches that of many food webs [51,54]. Third, the summary network's modularity (*Q* = 0.469) was within the range seen for many food webs (range 0.15–0.6) [64], suggesting that well-connected nodes were somewhat isolated and, again, restricted the effects of perturbations [52,65,66]. Overall, therefore, many structural aspects of the summary co-infection network suggested treatment or vaccination of a particular parasite may have little impact on the remaining network. This finding is consistent with treatment in human and wild rodent populations, where parasite populations rapidly return to pre-treatment levels, and secondary effects on other parasites are rarely reported [67,68]. Perturbation studies of parasite communities in other host species, more extensive monitoring of human treatment programmes, and dynamic co-infection networks are needed to more fully determine parasite community stability.

Resource- and immune-mediated indirect interactions between parasites were more common than expected by chance in the summary network. Co-infecting parasites tended to interact indirectly through shared resources rather than the immune system, and network modules tended to be associated with microhabitats rather than immune phenotypes. The dominance of indirect effects matched other ecological systems [69], and could be another reason why control programmes in co-infected populations rarely achieve eradication. The recognition of the dominance of resource-mediated relationships among co-infecting parasites, be they competition or facilitation, could lead to new, widely applicable metabolic therapies and broaden the importance of co-infection in the evolution of host–parasite interactions.

While much co-infection research has studied immune-mediated interactions [70], resource-mediated interactions have received less attention [71]. However, host resources are known to control the within-host dynamics of various individual parasite taxa: red blood cell density affects malaria intensity in laboratory mice and in humans [20,61], associations among microbiota [72], competitive exclusion of hepatitis or *Trypanosoma* strains [25,34] and the physiological location of parasites within nonhuman hosts [23,73]. Our results indicate that resources may be more widely involved in structuring parasite interactions in humans than currently appreciated. Such bottom-up control of the summary network could be produced by either facilitation or competition among parasites. In the case of facilitation, infection by one parasite encourages co-infection of the same resource, as with polymicrobial wound infection [74]. Conversely, ecological guilds of parasites may compete for particular resources [75]. We need further studies of the relative contributions of competition, facilitation, and how best to manipulate these interactions, to improve treatment of co-infected patients. If co-infecting parasites do predominantly interact via resources, then new treatments could be developed to disrupt co-infecting parasite populations that share resources. The apparent lesser influence of top-down immune control in the network suggests either that a strong immune response involving a few key components may prevent co-infection, or that components of the immune system are specialized, akin to specialist predators in free-living communities. The relative contribution of immune and resource control on co-infecting parasite populations needs further study.

As with any literature-derived data analysis, results may be influenced by observational and reporting biases [9]. We attempted to address these issues where possible (ESM, figures S2 and S7–S10). In the sampled publications, the number of parasite nodes and total nodes did not reach an asymptote, which suggests that parasites co-infecting humans are very diverse, with perhaps more than 200 other co-infecting parasites not included in our sample (ESM, figure S1*b*). The aspects of the summary network we study are robust to subsampling reviewed papers, and the fitted Michaelis–Menten curves suggest our summary network has captured most of the nodes. There may be detection or reporting biases in the sampled publications, for instance, because establishing immune mechanisms may be relatively more difficult in humans than *in vivo* experiments. Further research could identify whether individual networks assembled from particular co-infected patients are also resource-dominated, test for biomarkers of co-infection, and compare networks from different patients and points in the infection cycle to measure the health consequences of particular structures and dynamic states. Such focused efforts would also enable measurement of interaction strength, which would enable more sophisticated analyses such as probabilistic module detection, and prediction of treatment effects. Networks have much scope for improving treatment programmes [38].

Overall, we found that reported parasite interactions were most often indirect, a result that was robust to node aggregation and sampling of publications. It is therefore important to understand how treating one parasite species indirectly affects co-infecting parasites. Such indirect effects could be even more important than indicated by our analyses, given that we sampled only co-infecting parasites and interactions, and given the diversity and complexity of the commensal microbiome that our analyses did not include. Given the growing interest in integrated control strategies where multiple infections are treated simultaneously [5], we need to test whether knowledge of parasite interactions could improve treatment in human populations where co-infection is prevalent. While the complexity of the parasite community of humans makes this process somewhat daunting, knowing the patterns of interactions in the summary network presented herein makes this problem more tractable. With better understanding of the ecological interactions structuring parasite communities, the effects of treatment on the wider parasite community and on patient health could perhaps be predicted.

## Endnotes

1 NCI Enterprise Vocabulary Service (http://ncit.nci.nih.gov).

2 US National Library of Medicine (http://www.nlm.nih.gov/research/umls/META3_current_semantic_types.html).

## Data accessibility

The linked nodes in the summary network are included in a supplementary data file. The full database will be publicly available on Figshare.
